# (4,4′-Dimethyl-2,2′-bipyridine-κ^2^ *N*,*N*′)(dimethyl­formamide-κ*O*)diiodido­cadmium

**DOI:** 10.1107/S1600536812046648

**Published:** 2012-11-17

**Authors:** Sadif A. Shirvan, Sara Haydari Dezfuli, Fereydoon Khazali, Ali Borsalani

**Affiliations:** a Department of Chemistry, Omidieh Branch, Islamic Azad University, Omidieh, Iran; b Department of Petroleum Engineering, Omidieh Branch, Islamic Azad University, Omidieh, Iran

## Abstract

In the title compound, [CdI_2_(C_12_H_12_N_2_)(C_3_H_7_NO)], the Cd^II^ cation is five-coordinated in a distorted trigonal–bipyramidal configuration by two N atoms from a 4,4′-dimethyl-2,2′-bipyridine ligand, one O atom from a dimethyl­formamide ligand and two I^−^ anions. π–π stacking between pyridine rings of adjacent mol­ecules [centroid–centroid distance = 3.666 (3) and 3.709 (4) Å] stabilizes the three-dimensional structure

## Related literature

For related structures, see: Ahmadi *et al.* (2008 ▶); Alizadeh *et al.* (2010 ▶); Amani *et al.* (2009 ▶); Bellusci *et al.* (2008 ▶); Hojjat Kashani *et al.* (2008 ▶); Kalateh *et al.* (2008 ▶, 2010 ▶); Shirvan & Haydari Dezfuli (2012 ▶); Sofetis *et al.* (2006 ▶); Willett *et al.* (2001 ▶); Yousefi *et al.* (2008 ▶).
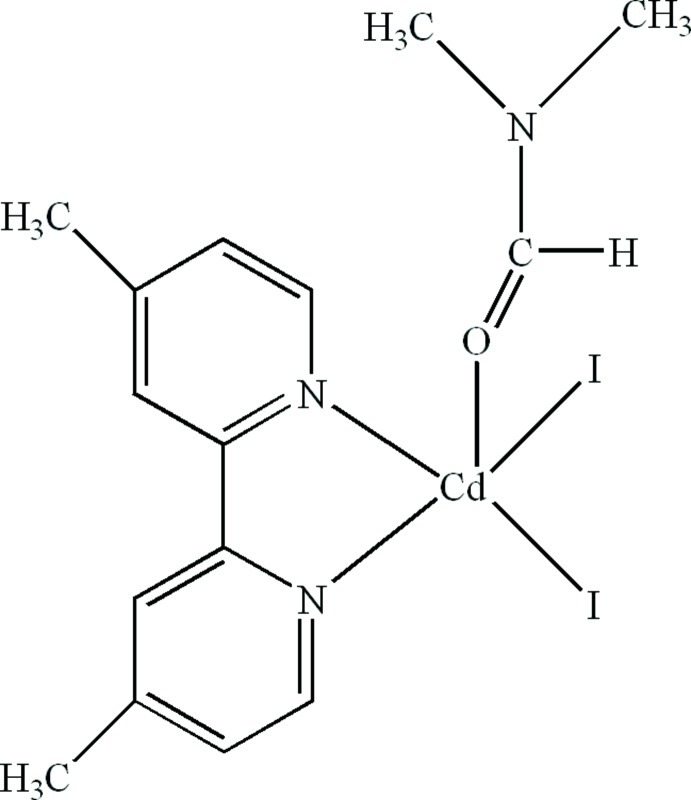


## Experimental

### 

#### Crystal data

[CdI_2_(C_12_H_12_N_2_)(C_3_H_7_NO)]
*M* *_r_* = 623.54
Monoclinic, 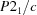

*a* = 8.6103 (6) Å
*b* = 15.1325 (8) Å
*c* = 15.4263 (10) Å
β = 98.347 (5)°
*V* = 1988.7 (2) Å^3^
*Z* = 4
Mo *K*α radiation
μ = 4.21 mm^−1^
*T* = 298 K
0.45 × 0.40 × 0.35 mm

#### Data collection

Bruker APEXII CCD area-detector diffractometer
Absorption correction: multi-scan (*SADABS*; Bruker, 2001 ▶) *T*_min_ = 0.188, *T*_max_ = 0.223
12059 measured reflections
3907 independent reflections
2859 reflections with *I* > 2σ(*I*)
*R*_int_ = 0.052

#### Refinement

*R*[*F* ^2^ > 2σ(*F* ^2^)] = 0.040
*wR*(*F* ^2^) = 0.084
*S* = 0.98
3907 reflections
199 parameters
H-atom parameters constrained
Δρ_max_ = 0.85 e Å^−3^
Δρ_min_ = −1.08 e Å^−3^

### 

Data collection: *APEX2* (Bruker, 2007 ▶); cell refinement: *SAINT* (Bruker, 2007 ▶); data reduction: *SAINT*; program(s) used to solve structure: *SHELXTL* (Sheldrick, 2008 ▶); program(s) used to refine structure: *SHELXTL*; molecular graphics: *SHELXTL*; software used to prepare material for publication: *SHELXTL*.

## Supplementary Material

Crystal structure: contains datablock(s) I, global. DOI: 10.1107/S1600536812046648/xu5650sup1.cif

Structure factors: contains datablock(s) I. DOI: 10.1107/S1600536812046648/xu5650Isup2.hkl

Additional supplementary materials:  crystallographic information; 3D view; checkCIF report

## Figures and Tables

**Table 1:** Selected bond lengths (Å)

Cd1—N1	2.327 (4)
Cd1—N2	2.365 (4)
Cd1—O1	2.345 (5)
Cd1—I1	2.7523 (7)
Cd1—I2	2.7635 (6)

**Fig. 1.**
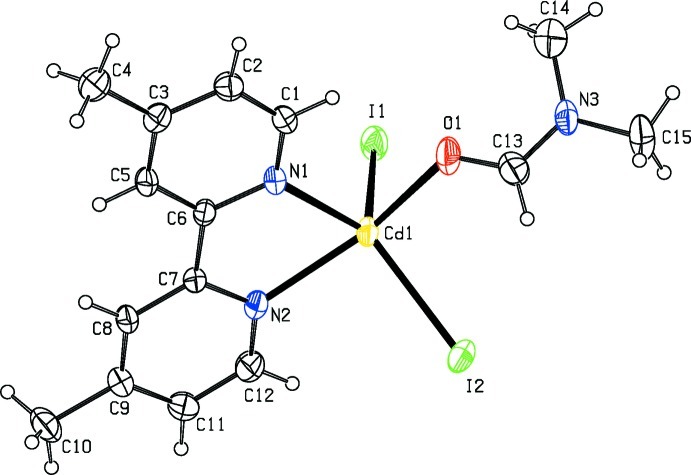
The molecular structure of the title molecule, with the atom-numbering scheme. Displacement ellipsoids are drawn at the 30% probability level.

**Fig. 2.**
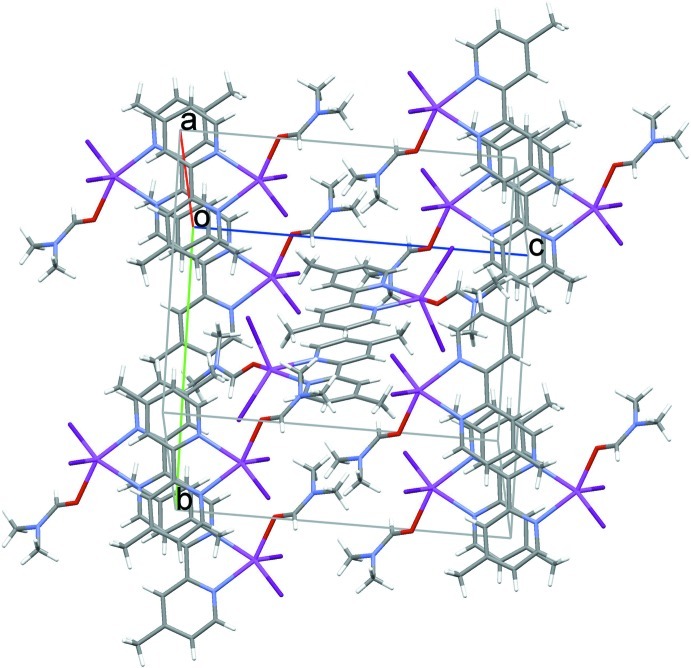
Unit-cell packing diagram for title molecule.

## supplementary crystallographic information

### Comment

Recently, we reported the synthes and crystal structure of [CdBr_2_(4,4'-dmbpy)(DMSO)] (Shirvan & Haydari Dezfuli, 2012) [where 4,4'-dmbpy is 4,4'-dimethyl-2,2'-bipyridine and DMSO is dimethyl sulfoxide]. 4,4'-Dimethyl-2,2'-bipyridine is a good bidentate ligand, and numerous complexes with 4,4'-dmbipy have been prepared, such as that of mercury (Kalateh *et al.*, 2008; Yousefi *et al.*, 2008), indium (Ahmadi *et al.*, 2008), iron (Amani *et al.*, 2009), platin (Hojjat Kashani *et al.*, 2008), silver (Bellusci *et al.*, 2008), gallium (Sofetis *et al.*, 2006), copper (Willett *et al.*, 2001), cadmium (Kalateh *et al.*, 2010) and zinc (Alizadeh *et al.*, 2010). Here, we report the synthesis and structure of the title compound.

In the title compound, (Fig. 1), the Cd^II^ atom is five-coordinated in a distorted trigonal-bipyramidal configuration by two N atoms from one 4,4'-dimethyl-2,2'-bipyridine, one O atom from one dimethylformamide and two I atoms. The Cd—N, Cd—I and Cd—O bond lengths and angles are collected in Table 1.

In the crystal structure, π-π contacts (Fig. 2) between the pyridine rings, *Cg*2—*Cg*2^i^ and *Cg*2—*Cg*3^ii^ [symmetry cods: (i) 1-*X*,-Y,-*Z* and (ii) 2-*X*,-Y,-*Z*, where *Cg*2 and *Cg*3 are centroids of the rings (N1/C1—C3/C5—C6) and (N2/C7—C9/C11—C12), respectively] stabilize the structure, with centroid-centroid distance of 3.666 (3) and 3.709 (4) Å.

### Experimental

For the preparation of the title compound, a solution of 4,4'-dimethyl-2,2'-bipyridine (0.15 g, 0.80 mmol) in methanol (10 ml) was added to a solution of CdI_2_, (0.29 g, 0.80 mmol) in methanol (5 ml) at room temperature. The suitable crystals for X-ray diffraction experiment were obtained by methanol diffusion to a colorless solution in dimethylformamide. Suitable crystals were isolated after one week (yield; 0.37 g, 74.2%).

### Refinement

H atoms were positioned geometrically with C—H = 0.93– 0.96 Å and constrained to ride on their parent atoms, with *U*_iso_(H) = 1.2*U*_eq_(C).

### Crystal data

**Table:**

[CdI_2_(C_12_H_12_N_2_)(C_3_H_7_NO)]	*F*(000) = 1168
*M**_r_* = 623.54	*D*_x_ = 2.083 Mg m^−^^3^
Monoclinic, *P*2_1_/*c*	Mo *K*α radiation, λ = 0.71073 Å
Hall symbol: -P 2ybc	Cell parameters from 12059 reflections
*a* = 8.6103 (6) Å	θ = 2.4–26.0°
*b* = 15.1325 (8) Å	µ = 4.21 mm^−^^1^
*c* = 15.4263 (10) Å	*T* = 298 K
β = 98.347 (5)°	Prism, colorless
*V* = 1988.7 (2) Å^3^	0.45 × 0.40 × 0.35 mm
*Z* = 4	

### Data collection

**Table:**

Bruker APEXII CCD area-detector diffractometer	3907 independent reflections
Radiation source: fine-focus sealed tube	2859 reflections with *I* > 2σ(*I*)
Graphite monochromator	*R*_int_ = 0.052
ω scans	θ_max_ = 26.0°, θ_min_ = 2.4°
Absorption correction: multi-scan (*SADABS*; Bruker, 2001)	*h* = −10→9
*T*_min_ = 0.188, *T*_max_ = 0.223	*k* = −18→17
12059 measured reflections	*l* = −19→19

### Refinement

**Table:**

Refinement on *F*^2^	Primary atom site location: structure-invariant direct methods
Least-squares matrix: full	Secondary atom site location: difference Fourier map
*R*[*F*^2^ > 2σ(*F*^2^)] = 0.040	Hydrogen site location: inferred from neighbouring sites
*wR*(*F*^2^) = 0.084	H-atom parameters constrained
*S* = 0.98	*w* = 1/[σ^2^(*F*_o_^2^) + (0.0426*P*)^2^] where *P* = (*F*_o_^2^ + 2*F*_c_^2^)/3
3907 reflections	(Δ/σ)_max_ = 0.007
199 parameters	Δρ_max_ = 0.85 e Å^−^^3^
0 restraints	Δρ_min_ = −1.08 e Å^−^^3^

### Special details

**Table:**

Geometry. All e.s.d.'s (except the e.s.d. in the dihedral angle between two l.s. planes) are estimated using the full covariance matrix. The cell e.s.d.'s are taken into account individually in the estimation of e.s.d.'s in distances, angles and torsion angles; correlations between e.s.d.'s in cell parameters are only used when they are defined by crystal symmetry. An approximate (isotropic) treatment of cell e.s.d.'s is used for estimating e.s.d.'s involving l.s. planes.
Refinement. Refinement of *F*^2^ against ALL reflections. The weighted *R*-factor *wR* and goodness of fit *S* are based on *F*^2^, conventional *R*-factors *R* are based on *F*, with *F* set to zero for negative *F*^2^. The threshold expression of *F*^2^ > σ(*F*^2^) is used only for calculating *R*-factors(gt) *etc*. and is not relevant to the choice of reflections for refinement. *R*-factors based on *F*^2^ are statistically about twice as large as those based on *F*, and *R*- factors based on ALL data will be even larger.

### Fractional atomic coordinates and isotropic or equivalent isotropic displacement parameters (Å^2^)

**Table:**

	*x*	*y*	*z*	*U*_iso_*/*U*_eq_	
C1	0.8789 (8)	−0.0736 (4)	0.0980 (3)	0.0488 (15)	
H1	0.9275	−0.0965	0.1510	0.059*	
C2	0.9026 (7)	−0.1170 (4)	0.0221 (4)	0.0477 (15)	
H2	0.9664	−0.1668	0.0242	0.057*	
C3	0.8286 (7)	−0.0843 (4)	−0.0571 (3)	0.0444 (14)	
C4	0.8494 (9)	−0.1285 (5)	−0.1415 (4)	0.0619 (19)	
H4A	0.8981	−0.0881	−0.1774	0.074*	
H4B	0.7488	−0.1460	−0.1719	0.074*	
H4C	0.9147	−0.1797	−0.1295	0.074*	
C5	0.7387 (7)	−0.0090 (4)	−0.0555 (3)	0.0404 (14)	
H5	0.6901	0.0149	−0.1080	0.049*	
C6	0.7189 (7)	0.0318 (4)	0.0225 (3)	0.0353 (12)	
C7	0.6243 (6)	0.1118 (4)	0.0260 (3)	0.0346 (12)	
C8	0.5479 (7)	0.1536 (4)	−0.0481 (3)	0.0412 (14)	
H8	0.5560	0.1305	−0.1031	0.049*	
C9	0.4610 (8)	0.2282 (4)	−0.0416 (4)	0.0500 (16)	
C10	0.3786 (10)	0.2740 (5)	−0.1238 (4)	0.070 (2)	
H10A	0.3051	0.2340	−0.1559	0.083*	
H10B	0.4550	0.2917	−0.1600	0.083*	
H10C	0.3238	0.3252	−0.1074	0.083*	
C11	0.4523 (8)	0.2614 (5)	0.0406 (4)	0.0552 (17)	
H11	0.3936	0.3118	0.0477	0.066*	
C12	0.5324 (8)	0.2183 (5)	0.1124 (4)	0.0546 (17)	
H12	0.5279	0.2417	0.1677	0.066*	
C13	1.0539 (9)	−0.0020 (5)	0.3557 (4)	0.0620 (19)	
H13	1.0829	0.0570	0.3635	0.074*	
C14	1.0840 (14)	−0.1519 (7)	0.4055 (6)	0.116 (4)	
H14A	1.1070	−0.1741	0.3504	0.140*	
H14B	0.9743	−0.1597	0.4087	0.140*	
H14C	1.1450	−0.1836	0.4525	0.140*	
C15	1.2436 (9)	−0.0323 (6)	0.4837 (4)	0.077 (3)	
H15A	1.2016	0.0111	0.5193	0.092*	
H15B	1.3308	−0.0074	0.4597	0.092*	
H15C	1.2782	−0.0828	0.5188	0.092*	
N1	0.7921 (6)	−0.0018 (3)	0.1005 (3)	0.0413 (12)	
N2	0.6153 (6)	0.1455 (3)	0.1065 (3)	0.0408 (12)	
N3	1.1228 (6)	−0.0590 (4)	0.4127 (3)	0.0526 (14)	
O1	0.9541 (6)	−0.0201 (4)	0.2930 (3)	0.0708 (15)	
Cd1	0.74180 (5)	0.06619 (3)	0.22889 (2)	0.04265 (13)	
I1	0.51473 (6)	−0.03208 (3)	0.29304 (2)	0.05923 (15)	
I2	0.84760 (6)	0.21673 (3)	0.32035 (3)	0.06213 (16)	

### Atomic displacement parameters (Å^2^)

**Table:**

	*U*^11^	*U*^22^	*U*^33^	*U*^12^	*U*^13^	*U*^23^
C1	0.066 (4)	0.042 (4)	0.035 (3)	0.010 (3)	−0.003 (3)	0.003 (2)
C2	0.051 (4)	0.045 (4)	0.047 (3)	0.010 (3)	0.006 (3)	−0.002 (3)
C3	0.046 (3)	0.047 (4)	0.041 (3)	−0.004 (3)	0.008 (2)	−0.006 (2)
C4	0.082 (5)	0.059 (5)	0.047 (3)	0.011 (4)	0.016 (3)	−0.012 (3)
C5	0.049 (4)	0.046 (4)	0.025 (2)	0.001 (3)	0.004 (2)	0.001 (2)
C6	0.038 (3)	0.039 (3)	0.028 (2)	−0.006 (3)	0.001 (2)	−0.001 (2)
C7	0.040 (3)	0.034 (3)	0.029 (2)	−0.002 (3)	0.001 (2)	0.001 (2)
C8	0.052 (4)	0.043 (4)	0.026 (2)	0.004 (3)	0.000 (2)	0.002 (2)
C9	0.062 (4)	0.047 (4)	0.038 (3)	0.002 (3)	−0.003 (3)	0.007 (2)
C10	0.090 (6)	0.066 (5)	0.050 (3)	0.028 (4)	0.000 (3)	0.015 (3)
C11	0.063 (4)	0.044 (4)	0.057 (4)	0.015 (3)	0.000 (3)	−0.001 (3)
C12	0.069 (5)	0.052 (4)	0.043 (3)	0.008 (4)	0.007 (3)	−0.009 (3)
C13	0.063 (5)	0.062 (5)	0.057 (4)	−0.001 (4)	−0.004 (3)	0.011 (3)
C14	0.162 (11)	0.083 (7)	0.085 (6)	−0.012 (7)	−0.044 (7)	0.003 (5)
C15	0.067 (5)	0.115 (7)	0.042 (3)	−0.008 (5)	−0.012 (3)	0.009 (4)
N1	0.048 (3)	0.044 (3)	0.029 (2)	0.001 (2)	−0.0041 (19)	0.0007 (19)
N2	0.047 (3)	0.044 (3)	0.031 (2)	0.000 (2)	0.0004 (19)	−0.0054 (19)
N3	0.058 (3)	0.067 (4)	0.029 (2)	0.004 (3)	−0.003 (2)	0.000 (2)
O1	0.077 (4)	0.077 (4)	0.048 (2)	0.011 (3)	−0.025 (2)	−0.007 (2)
Cd1	0.0527 (3)	0.0474 (3)	0.02556 (18)	−0.0051 (2)	−0.00212 (15)	−0.00121 (16)
I1	0.0755 (3)	0.0669 (3)	0.03425 (19)	−0.0214 (2)	0.00443 (18)	0.00662 (17)
I2	0.0806 (3)	0.0571 (3)	0.0446 (2)	−0.0157 (2)	−0.0047 (2)	−0.01291 (18)

### Geometric parameters (Å, º)

**Table:**

C1—N1	1.322 (8)	C10—H10C	0.9600
C1—C2	1.383 (8)	C11—C12	1.380 (8)
C1—H1	0.9300	C11—H11	0.9300
C2—C3	1.385 (8)	C12—N2	1.323 (8)
C2—H2	0.9300	C12—H12	0.9300
C3—C5	1.379 (9)	C13—O1	1.229 (8)
C3—C4	1.498 (8)	C13—N3	1.310 (8)
C4—H4A	0.9600	C13—H13	0.9300
C4—H4B	0.9600	C14—N3	1.445 (11)
C4—H4C	0.9600	C14—H14A	0.9600
C5—C6	1.384 (7)	C14—H14B	0.9600
C5—H5	0.9300	C14—H14C	0.9600
C6—N1	1.373 (6)	C15—N3	1.454 (8)
C6—C7	1.464 (8)	C15—H15A	0.9600
C7—N2	1.355 (6)	C15—H15B	0.9600
C7—C8	1.386 (7)	C15—H15C	0.9600
C8—C9	1.367 (9)	Cd1—N1	2.327 (4)
C8—H8	0.9300	Cd1—N2	2.365 (4)
C9—C11	1.377 (9)	Cd1—O1	2.345 (5)
C9—C10	1.527 (8)	Cd1—I1	2.7523 (7)
C10—H10A	0.9600	Cd1—I2	2.7635 (6)
C10—H10B	0.9600		
			
N1—C1—C2	124.6 (5)	N2—C12—C11	123.4 (5)
N1—C1—H1	117.7	N2—C12—H12	118.3
C2—C1—H1	117.7	C11—C12—H12	118.3
C1—C2—C3	118.1 (6)	O1—C13—N3	125.3 (7)
C1—C2—H2	120.9	O1—C13—H13	117.3
C3—C2—H2	120.9	N3—C13—H13	117.3
C5—C3—C2	117.8 (5)	N3—C14—H14A	109.5
C5—C3—C4	121.6 (5)	N3—C14—H14B	109.5
C2—C3—C4	120.6 (6)	H14A—C14—H14B	109.5
C3—C4—H4A	109.5	N3—C14—H14C	109.5
C3—C4—H4B	109.5	H14A—C14—H14C	109.5
H4A—C4—H4B	109.5	H14B—C14—H14C	109.5
C3—C4—H4C	109.5	N3—C15—H15A	109.5
H4A—C4—H4C	109.5	N3—C15—H15B	109.5
H4B—C4—H4C	109.5	H15A—C15—H15B	109.5
C3—C5—C6	121.7 (5)	N3—C15—H15C	109.5
C3—C5—H5	119.2	H15A—C15—H15C	109.5
C6—C5—H5	119.2	H15B—C15—H15C	109.5
N1—C6—C5	119.9 (5)	C1—N1—C6	117.9 (5)
N1—C6—C7	117.4 (4)	C1—N1—Cd1	124.4 (3)
C5—C6—C7	122.7 (5)	C6—N1—Cd1	117.6 (4)
N2—C7—C8	120.0 (5)	C12—N2—C7	118.7 (5)
N2—C7—C6	116.8 (4)	C12—N2—Cd1	123.9 (4)
C8—C7—C6	123.1 (5)	C7—N2—Cd1	117.4 (4)
C9—C8—C7	121.1 (5)	C13—N3—C14	120.8 (6)
C9—C8—H8	119.5	C13—N3—C15	121.7 (7)
C7—C8—H8	119.5	C14—N3—C15	117.4 (6)
C8—C9—C11	118.2 (5)	C13—O1—Cd1	128.4 (5)
C8—C9—C10	120.5 (5)	N1—Cd1—O1	83.23 (16)
C11—C9—C10	121.3 (6)	N1—Cd1—N2	70.51 (16)
C9—C10—H10A	109.5	O1—Cd1—N2	149.27 (18)
C9—C10—H10B	109.5	N1—Cd1—I1	107.25 (13)
H10A—C10—H10B	109.5	O1—Cd1—I1	95.65 (14)
C9—C10—H10C	109.5	N2—Cd1—I1	106.98 (12)
H10A—C10—H10C	109.5	N1—Cd1—I2	135.29 (13)
H10B—C10—H10C	109.5	O1—Cd1—I2	93.72 (12)
C9—C11—C12	118.7 (6)	N2—Cd1—I2	93.95 (12)
C9—C11—H11	120.7	I1—Cd1—I2	117.42 (2)
C12—C11—H11	120.7		
			
N1—C1—C2—C3	−1.1 (11)	C6—C7—N2—C12	179.2 (6)
C1—C2—C3—C5	1.5 (9)	C8—C7—N2—Cd1	177.8 (4)
C1—C2—C3—C4	−179.9 (6)	C6—C7—N2—Cd1	−3.2 (6)
C2—C3—C5—C6	−1.6 (9)	O1—C13—N3—C14	−0.2 (13)
C4—C3—C5—C6	179.8 (6)	O1—C13—N3—C15	178.0 (7)
C3—C5—C6—N1	1.1 (9)	N3—C13—O1—Cd1	147.5 (6)
C3—C5—C6—C7	179.9 (6)	C1—N1—Cd1—O1	16.0 (5)
N1—C6—C7—N2	−0.8 (8)	C6—N1—Cd1—O1	−168.1 (4)
C5—C6—C7—N2	−179.6 (5)	C1—N1—Cd1—N2	179.8 (6)
N1—C6—C7—C8	178.2 (5)	C6—N1—Cd1—N2	−4.4 (4)
C5—C6—C7—C8	−0.6 (9)	C1—N1—Cd1—I1	−77.8 (5)
N2—C7—C8—C9	−0.9 (9)	C6—N1—Cd1—I1	98.0 (4)
C6—C7—C8—C9	−179.9 (6)	C1—N1—Cd1—I2	104.5 (5)
C7—C8—C9—C11	0.6 (10)	C6—N1—Cd1—I2	−79.7 (4)
C7—C8—C9—C10	179.5 (6)	C13—O1—Cd1—N1	146.2 (7)
C8—C9—C11—C12	0.5 (11)	C13—O1—Cd1—N2	115.2 (6)
C10—C9—C11—C12	−178.4 (7)	C13—O1—Cd1—I1	−107.0 (6)
C9—C11—C12—N2	−1.3 (11)	C13—O1—Cd1—I2	11.0 (6)
C2—C1—N1—C6	0.6 (10)	C12—N2—Cd1—N1	−178.6 (6)
C2—C1—N1—Cd1	176.4 (5)	C7—N2—Cd1—N1	4.0 (4)
C5—C6—N1—C1	−0.6 (8)	C12—N2—Cd1—O1	−145.7 (5)
C7—C6—N1—C1	−179.4 (6)	C7—N2—Cd1—O1	36.9 (6)
C5—C6—N1—Cd1	−176.7 (4)	C12—N2—Cd1—I1	78.7 (5)
C7—C6—N1—Cd1	4.5 (6)	C7—N2—Cd1—I1	−98.8 (4)
C11—C12—N2—C7	0.9 (10)	C12—N2—Cd1—I2	−41.6 (5)
C11—C12—N2—Cd1	−176.5 (5)	C7—N2—Cd1—I2	140.9 (4)
C8—C7—N2—C12	0.2 (9)		
